# Application of PBL-CBL and Mini-CEX methods in the standardized training of residents in nephrology department: A prospective study

**DOI:** 10.12669/pjms.40.9.9434

**Published:** 2024-10

**Authors:** Xiaodong Li

**Affiliations:** Xiaodong Li, Department of Nephrology, Baoding No.1 Central Hospital of Hebei Medical University, Baoding 071000, Hebei, China

**Keywords:** Standardized training for residents, Problem-based learning, Case-based learning, Mini-clinical evaluation exercise

## Abstract

**Objective:**

This study explores the application and feasibility of problem based learning (PBL), integrating case based learning (CBL), and the mini clinical evaluation exercise (Mini-CEX) into the standardized training of residents in nephrology department, in order to assess their clinical skills in a comprehensive manner.

**Methods:**

This prospective study enrolled 60, three years residents majoring in clinical medicine, from June 2022 to December 2023 in Nephrology Department of Baoding No.1 Central Hospital. These participants were randomly allocated into either the combined PBL-CBL and Mini-CEX teaching group (experimental group) or the traditional lecture-based teaching group (control group). Two groups were evaluated with Mini-CEX assessments and test scores upon exit the department, followed by a questionnaire survey to measure satisfaction levels for the teachers.

**Results:**

There was no statistically significant difference in age, sex, year of graduate school, specialty and student source between the two groups of residents upon the entering the department (P>0.05). Upon the completion, the experimental group, which received PBL-CBL and Mini-CEX teaching methods, demonstrated significantly higher scores in all aspects compared to the control group, with a statistically significant difference (P<0.01). Furthermore, compared the control group, most residents in the experimental group agreed that the PBL-CBL and Mini-CEX teaching methods improve curiosity and enthusiasm for learning (73.3%), communication and expression abilities (73.3%), self-learning abilities (80%), understanding of diseases (76.7%), and like this teaching method (86.7%).

**Conclusions:**

PBL–CBL and Mini-CEX may be an effective method for improving medical residents’ performance and enhancing their clinical skills, which is worthy of promotion in the standardized training of the residents.

## INTRODUCTION

The standardized training for residents (STR) focuses on providing clinical practice, professional theoretical knowledge, political ideology, and professional ethics training for graduates.^1^ STR serves as a crucial transition stage for medical students to become doctors after graduation, playing an important bridging role in post-graduate medical education. STR provides comprehensive, systematic, and rigorous clinical teaching and practice to ensure that trainee physicians fully grasp the relevant professional knowledge, including basic theory, skills, and the diagnosis and treatment of common diseases.^2^

Due to lots of sub-specialties, the training of internal medicine residents requires rotation through numerous departments, with very limited time in each specialty. Additionally, kidney disease is highly comprehensive and closely linked to pathology and physiology, making it difficult for residents to fully grasp within a short period of time. Hence, finding a solution to enable residents to acquire specialized knowledge and skills within the limited rotation period is an urgent challenge.

The traditional teaching model, which is led by preceptor teachers and adopts a classroom-based theoretical indoctrination method, is no longer suitable for the training of modern medical students. Many medical universities both domestically and internationally have begun to explore teaching reforms.^3^-^5^ Problem-based learning (PBL) is a discussion-based instructional approach centered around real-world problems. Its aim is to cultivate students’ ability to actively learn and analyze problems, thereby enabling them to master clinical knowledge and specialized skills. PBL also contributes to enhancing students’ subjective initiative, enthusiasm for learning, and their ability to apply theoretical knowledge to solve specific clinical problems. Case-based learning (CBL), on the other hand, is an instructional method that revolves around concrete clinical cases, involving the design of a series of related questions based on these cases that guide students to engage in active discussion and analysis. The mini-clinical evaluation exercise (Mini-CEX) serves as an assessment tool for evaluating the clinical capabilities of residents, while also functioning as an educational tool.^6^ It features the distinctive characteristics of direct observation and timely feedback, ultimately enhancing the comprehensive abilities of residents in clinical practice.

This study discusses how to better conduct clinical teaching in the nephrology department during the STR phase. It explores the actual effects of combining PBL, CBL, and Mini-CEX teaching methods in the STR process, and attempts to explore a new teaching model in specific clinical STR practices to improve teaching quality.

## METHODS

A total of 60 residents who underwent a two months standardized training in the Nephrology Department of Baoding No.1 Central Hospital from June 2022 to September 2023 were selected as the research subjects, with 30 residents in the control group and 30 ones in the experimental group. Random drawing was used to assign each batch of residents to their respective groups upon admission, and there were no statistically significant differences in age, sex, year of graduate school, specialty, and student source between the two groups (*P*>0.05, Table-I), indicating their comparability. The teachers were all clinical doctors with a senior physician title or above and more than three years of clinical teaching experience, possessing enthusiasm and qualifications, who have undergone relevant training for residents in our hospital and obtained the certification. Our study was approved by Ethics Committee of Baoding No.1 Central Hospital. Reference number: 2022-045.

**Table-I:** Comparison of baseline data for all residents between the two groups.

Variables	Con (n = 30)	Exp (n = 30)	Statistic	P
Age (years)	26.27 ± 1.72	25.70 ± 1.80	t=1.245	0.218
Gender, n (%)			χ²=0.089	0.766
Female	23 (76.67)	22 (73.33)		
Male	7 (23.33)	8 (26.67)		
Year of graduate, n (%)			χ²=0.890	0.641
First	14 (46.67)	17 (56.67)		
Second	7 (23.33)	7 (23.33)		
Third	9 (30.00)	6 (20.00)		
Specialty, n (%)			-	0.542
Internal medicine	24	26		
Critical medicine	4	2		
Laboratory medicine	2	2		
Resident source, n (%)			-	0.477
Professional postgraduate	18 (60.00)	22 (73.33)		
Institute resident	6 (20.00)	5 (16.67)		
Social resident	6 (20.00)	3 (10.00)		

The Mini-CEX assessment is used to quantitatively score the capabilities of residents, encompassing seven aspects including medical history inquiring, physical examination, humanistic care, clinical assessment, health education consultation, organizational efficiency, and overall performance.^7^ The scoring is based on a 9-point scale, with 1-3 points indicating unsatisfactory performance, 4-6 points indicating satisfactory performance, and 7-9 points indicative of excellent performance. Patients of nephrotic syndrome, diabetic nephropathy, chronic kidney disease, acute kidney injury, and urinary tract infection are selected as the Mini-CEX evaluation cases, with all those being in stable condition during the evaluations.

### Teaching Methods:

Traditional teaching method is used in control group, with teachers instructing residents in patient diagnosis, doctor-patient communication, medical record writing, and daily ward rounds. Residents in the group imitate or self-study during this process. While in experimental group, PBL-CBL and Mini-CEX teaching methods are incorporated into daily clinical work, providing real-time assessment and timely feedback to residents during the teaching process. Assess the effectiveness of various teaching approaches based on the following three criteria. The examination was conducted by two instructors in the nephrology department. The identities of the students and their respective groups will remain undisclosed to the assessors.

### Teaching effect evaluation:

### Comprehensive competency evaluation:

All residents are assessed upon exit the department, with tests based on the teaching outline and clinical practical needs. The content includes theoretical test, case analysis, and skill operation. The theoretical test carries a maximum of 100 points and is comprised solely of objective questions. The case analysis is also worth 100 points and focuses on a nephrotic syndrome case. The skill operation, also worth 100 points, encompasses urinary catheterization and cardiopulmonary resuscitation.

### Mini-CEX assessment:

A designated teacher adopts the Mini-CEX evaluation form to assess the residents upon the completion.

### Satisfaction evaluation:

All residents fill out a questionnaire survey for their satisfaction with the teaching methods and effects. The survey includes five items: stimulate curiosity and enthusiasm for learning, refine abilities in communication and expression, improve self-learning ability, enhance understanding of diseases, and like this teaching method. The residents completed a table by indicating “yes” or “no” for each item in the questionnaire, based on their personal perceptions of how the class had enhanced their different abilities and their overall enjoyment of the teaching.

### Statistical methods:

The analysis of the students’ attitudes towards teaching methods in the two groups was conducted using Pearson’s chi-squared test. The mean scores for the theoretical test, case analysis, skill operation and each Mini-CEX evaluation were presented with their respective standard deviations (SD). The Shapiro-Wilk test was utilized to assess the normal distribution of the data. A comparison between different groups was performed using an independent sample t-test. All statistical tests were two-sided, with significance set at *p* < 0.05. Data analysis was carried out by GraphPadPrism 8.0.

## RESULTS

### Comparison of baseline data for all residents between the two groups:

All 60 residents completed the assessments and surveys after an eight weeks rotation. The experimental group consisted of 30 residents, comprising eight males and 22 females, while the control group also consisted of 30 ones, with seven males and 23 females. Upon entering the department, there were no statistically significant differences in age, gender, year of graduate, specialty, or resident source between the two groups of residents (*P*>0.05, Table-I).

**Fig.1:**
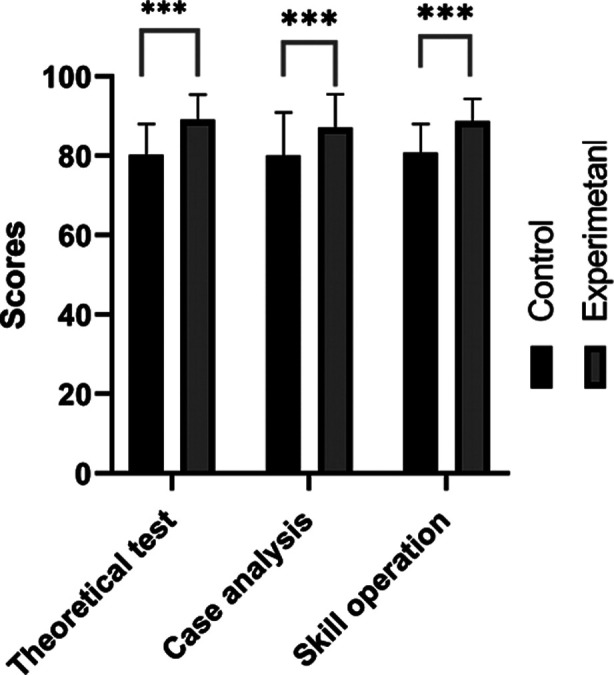
The average scores for the theoretical test, case analysis, and skill operation for all participants in the two groups.

Comparison of average scores for the theoretical test, case analysis, and skill operation for all participants between the two groups. The average scores for the theoretical test, case analysis, and skill operation for all participants are presented in Fig.1. Comparatively, the experimental group demonstrated notably higher average scores for the theoretical test, case analysis, and skill operation as compared to the control group *(P*<0.01, Table-II).

**Table-II:** Comparison of average scores for the theoretical test, case analysis, and skill operation for all participants.

Variables	Con (n = 30)	Exp (n = 30)	Statistic	P
Theoretical test scores	80.17 ± 7.91	89.10 ± 6.32	t=-4.834	<0.001
Case analysis scores	80.10 ± 10.83	87.10 ± 8.46	t=-2.790	0.007
Skill operation scores	80.83 ± 7.18	88.73 ± 5.64	t=-4.739	<0.001

Comparison of the average scores for each Mini-CEX evaluation among all participants exiting the department in both groups. Upon completion, the mean scores of each Mini-CEX assessment for the experimental group residents were found to be obviously higher than those of the control group, with statistically significant differences (*P* < 0.01) as shown in Table-III.

**Table-III:** Comparison of the average scores for each Mini-CEX evaluation among all participants exiting the department.

	Con (n = 30)	Exp (n = 30)	Statistic	p
Medical history inquiring	5.93 ± 1.53	7.17 ± 1.34	t=-3.320	0.002
Physical examination	5.70 ± 1.56	7.77 ± 1.30	t=-5.572	<0.001
Humanistic care	5.57 ± 1.57	7.83 ± 1.15	t=-6.388	<0.001
Clinical assessment	5.97 ± 1.52	8.13 ± 0.86	t=-6.796	<0.001
Health education consultation	5.67 ± 1.27	7.07 ± 1.46	t=-3.964	<0.001
Organization efficiency	6.03 ± 1.43	7.77 ± 1.30	t=-4.912	<0.001
Overall performance	6.60 ± 1.45	7.97 ± 1.00	t=-4.245	<0.001

Comparison of satisfaction with the teaching methods among all residents in the two groups. In comparison to the control group, a majority of participants in the experimental group indicated a favorable response towards the PBL-CBL and Mini-CEX teaching methods. Specifically, 73.3% agreed that these methods improved their curiosity and enthusiasm for learning, 73.3% believed it enhanced their communication and expression abilities, 80% reported an improvement in their self-learning abilities, 76.7% indicated better understanding of diseases, and 86.7% expressed a preference for this teaching approach, with statistically significant differences (*P*<0.01) (Table-IV).

**Table-IV:** Comparison of satisfaction with the teaching methods among all residents.

Variables	Con (n = 30)	Exp (n = 30)	Statistic	P
Stimulate curiosity and enthusiasm for learning, n (%)			χ²=5.554	0.018
No	17 (56.67)	8 (26.67)		
Yes	13 (43.33)	22 (73.33)		
Refine abilities in communication and expression, n (%)			χ²=8.148	0.004
No	19 (63.33)	8 (26.67)		
Yes	11 (36.67)	22 (73.33)		
Improve self learning ability, n (%)			χ²=17.143	<0.001
No	22 (73.33)	6 (20.00)		
Yes, n(%)	8 (26.67)	24 (80.00)		
Enhance understanding of diseases, n (%)			χ²=13.125	<0.001
No	21 (70.00)	7 (23.33)		
Yes	9 (30.00)	23 (76.67)		
Like this teaching method, n (%)			χ²=17.778	<0.001
No	20 (66.67)	4 (13.33)		
Yes	10 (33.33)	26 (86.67)		

***Note:*** *** <0.001.

## DISCUSSION

This study combined the PBL-CBL with Mini-CEX teaching methods and applied them to the nephrology STR. The findings showed that the experimental group, which received PBL-CBL and Mini-CEX teaching methods, achieved significantly higher scores in all aspects compared to the control group. Additionally, a majority of residents in the experimental group agreed that the PBL-CBL and Mini-CEX teaching methods enhanced curiosity and enthusiasm for learning, communication and expression abilities, self-learning abilities, understanding of diseases, and overall satisfaction with this teaching approach.

STR focuses on the goal of transitioning medical graduates from the classroom to the bedside, applying their medical knowledge to solve specific clinical problems, and continuously improving their clinical reasoning abilities in clinical practice. Therefore, the requirements for training are higher than those for interns. Nephrology is an important component of internal medicine and a highly specialized core course. Kidney diseases pose learning challenges in standardized residency training for physicians, as they often have few clinical signs, complex pathophysiology, and easily confusable pathological types. Traditional teaching methods are often dull and abstract, leading to low student engagement. Therefore, there is a new challenge in improving the STR in nephrology, which presents new challenges for clinical teaching in nephrology.

Many medical universities have begun to explore educational reforms, including teaching methods such as CBL, PBL, mini-CEX, evidence based medicine, internet-based teaching, and simulation teaching.^8^-^10^ However, each teaching method has its pros and cons, so effectively combining them to complement each other has been a focus of attention and exploration in recent years. CBL and PBL are two commonly used teaching methods, with the combination of both being widely applied, while mini-CEX has also been effectively used on its own. In our study, we are attempting for the first time to combine PBL-CBL with Mini-CEX for application in nephrology teaching in STR.

PBL is a problem-based learning method, where residents cultivate problem-solving and teamwork skills through self-directed learning and collaborative problem-solving.^11^-^13^ In this study, the teachers presented a challenging common clinical problem in nephrology for students to solve together in groups. Residents were required to independently gather and analyze information, and then engaged in group discussions and collaboration to find the best solutions. CBL is a case-based learning method, where students develop knowledge and problem-solving skills through the analysis and discussion of real or virtual cases.^14^ In this study, the teachers designed a series of common nephrology-related cases for residents to study and discuss, in accordance with their curriculum. Residents can enhance their clinical reasoning and decision-making abilities by working together in groups to discuss the diagnosis and treatment plans for the cases. The traditional mentoring model emphasizes the learning and training of students in actual clinical practice, relying mainly on experienced mentors.

This reliance on mentors can impact the quality and effectiveness of training due to variations in their levels of expertise and teaching styles. The training process heavily depends on direct observation and feedback from mentors, lacking standardized assessment tools and methods. Mini-CEX is a clinical assessment tool used to evaluate the performance of medical students or residents in clinical practice.^15^ It is based on the principle of direct observation and feedback, providing personalized guidance and improvement opportunities through the assessment of students’ actual clinical practice. This study found that the Mini-CEX can more directly identify deficiencies in resident’s clinical diagnosis and treatment, provide specific and real-time feedback to residents after assessment, discover problems in the entire teaching process, make timely adjustments to teaching strategies, improve learning methods, and explore more suitable teaching strategies to enhance students’ clinical abilities.

This study has found that combining the PBL-CBL with Mini-CEX teaching methods has many advantages: firstly, it allows for a comprehensive assessment of residents’ clinical practice abilities, problem-solving skills, teamwork, and self-directed learning abilities. This helps in identifying residents’ strengths and weaknesses, and in developing personalized training plans. Secondly, the PBL-CBL teaching method emphasizes residents’ ability to learn and solve problems in real-life situations, while the Mini-CEX evaluation provides a direct assessment of learners’ application of knowledge and skills in clinical practice, bridging the gap between theory and practice.^16^-^18^ This helps residents improve their clinical reasoning and decision-making abilities.After reviewing the relevant literature, this study represents the first application of utilizing PBL-CBL and Mini-CEX methods in standardized training.

### Limitations:

Firstly, despite the similarity in teaching content between the two groups, there were slight variations in the teaching methods utilized. As a result, it was challenging to determine whether these differences had any impact on the outcomes. Additionally, both groups had the same lead teacher for their courses, which was intended to prevent bias stemming from variations in teaching levels.

This meant that it was not possible to implement double-blinding, potentially affecting the credibility of the findings. It is also worth noting that residents in the experimental group appeared to allocate more time to self-study after class, a factor that was not accurately measured in the experimental design and could potentially skew the efficacy results. Additionally, this study was conducted at a single center and had a relatively small sample size, indicating that the superiority of the model would benefit from additional randomized controlled data from various departments across multiple teaching hospitals. Therefore, the conclusions require further validation through multicenter prospective studies and the involvement of more residents in the field. Lastly, the study was conducted in China, so its suitability for residents in other countries also needs to be taken into consideration. Hence, further investigation is necessary to validate the results in diverse populations and environments.

## CONCLUSIONS

The reasonable application of teaching methods and assessment systems for STR requires continuous experimentation and exploration. The application of PBL-CBL and Mini-CEX methods in the nephrology STR is more beneficial for cultivating comprehensive abilities in medical residents, such as proactive learning, independent thinking, effective communication, and teamwork. It also helps residents apply their knowledge to analyze and solve real clinical cases, laying a solid foundation for their future medical careers. This teaching model is worth further exploration and application.

## Data availability statement:

The original contributions presented in the study are included in the article materials, further inquiries can be directed to the corresponding author.

## References

[1] Xiao Y, Zhu SY (2020). China will fully implement the standardised training system for residents in 2020. Postgrad Med J.

[2] He Y, Qian W, Shi L, Zhang K, Huang J (2020). Standardized residency training:An equalizer for residents at different hospitals in Shanghai, China?. Int J Health Plann Manage.

[3] Ali SMH, Masood HMU, Malik A (2022). Need for a single standardized licensing &residency-entrance level exam policy in the medical education system of Pakistan. Pak J Med Sci.

[4] Sekar DR, Siropaides CH, Smith LN, Nguyen OK (2021). Adapting Existing Resources for Serious Illness Communication Skills Training for Internal Medicine Residents. South Med J.

[5] Kapadia MR, Lee E, Healy H, Dort JM, Rosenbaum ME, Newcomb AB (2021). Training Surgical Residents to Communicate with Their Patients:A Scoping Review of the Literature. J Surg Educ.

[6] Niu L, Mei Y, Xu X, Guo Y, Li Z, Dong S (2022). A novel strategy combining Mini-CEX and OSCE to assess standardized training of professional postgraduates in department of prosthodontics. BMC Med Educ.

[7] Johnson NR, Pelletier A, Berkowitz LR (2020). Mini-Clinical Evaluation Exercise in the Era of Milestones and Entrustable Professional Activities in Obstetrics and Gynaecology:Resume or Reform?. J Obstet Gynaecol Can.

[8] Zhao W, He L, Deng W, Zhu J, Su A, Zhang Y (2020). The effectiveness of the combined problem-based learning (PBL) and case-based learning (CBL) teaching method in the clinical practical teaching of thyroid disease. BMC Med Educ.

[9] Wang A, Xiao R, Zhang C, Yuan L, Lin N, Yan L (2022). Effectiveness of a combined problem-based learning and flipped classroom teaching method in ophthalmic clinical skill training. BMC Med Educ.

[10] Zeri F, Eperjesi F, Woods C, Bandlitz S, Kumar Bhootra A, Joshi MR (2023). Evidence-based teaching in contact lenses education:Teaching and learning strategies. Cont Lens Anterior Eye.

[11] Trullàs JC, Blay C, Sarri E, Pujol R (2022). Effectiveness of problem-based learning methodology in undergraduate medical education:a scoping review. BMC Med Educ.

[12] Hydrie MZI, Naqvi SMZH, Alam SN, Jafry SIA (2021). Kolb's Learning Style Inventory 4.0 and its association with traditional and problem based learning teaching methodologies in medical students. Pak J Med Sci.

[13] Arain SA, Alhadid DA, Rasheed S, Alrefaai MM, Alsibai TMA, Meo SA (2021). Perceived effectiveness of learning methods among preclinical medical students - role of personality and changes over time. Pak J Med Sci.

[14] Cen XY, Hua Y, Niu S, Yu T (2021). Application of case-based learning in medical student education:a meta-analysis. Eur Rev Med Pharmacol Sci.

[15] He Y, Wen S, Zhou M, Li X, Gong M, Zhou L (2022). A Pilot Study of Modified Mini-Clinical Evaluation Exercises (Mini-CEX) in Rotation Students in the Department of Endocrinology. Diabetes Metab Syndr Obes.

[16] Yang W, Li H, Su A, Ding L (2023). Application of problem based learning (PBL) and case based learning (CBL) in the teaching of international classification of diseases encoding. Sci Rep.

[17] Wang H, Xuan J, Liu L, Shen X, Xiong Y (2021). Problem-based learning and case-based learning in dental education. Ann Transl Med.

[18] Liang Y, Noble LM (2021). Chinese doctors'views on workplace-based assessment:trainee and supervisor perspectives of the mini-CEX. Med Educ Online.

